# Perceived Barriers and Facilitators of Implementing a Multicomponent Intervention to Improve Communication With Older Adults With and Without Dementia (SHARING Choices) in Primary Care: A Qualitative Study

**DOI:** 10.1177/21501319221137251

**Published:** 2022-11-18

**Authors:** Kelly M. Smith, Danny Scerpella, Amy Guo, Naaz Hussain, Jessica L. Colburn, Valerie T. Cotter, Jennifer Aufill, Sydney M. Dy, Jennifer L. Wolff

**Affiliations:** 1 Michael Garron Hospital – Toronto East Health Network, Toronto, ON, Canada; 2 University of Toronto, Toronto, ON, Canada; 3 Johns Hopkins Bloomberg School of Public Health, Baltimore, MD, USA; 4 Johns Hopkins Medicine, Frederick, MD, USA; 5 Johns Hopkins School of Medicine, Baltimore, MD, USA; 6 Johns Hopkins School of Nursing, Baltimore, MD, USA

**Keywords:** dementia, end of life, geriatrics, patient-centeredness, practice management, primary care, qualitative methods

## Abstract

**Introduction:**

Implementing patient- and family-centered communication strategies has proven challenging in primary care, particularly for persons with dementia. To address this, we designed SHARING Choices, a multicomponent intervention combining patient and family partnered agenda setting, electronic portal access, and supports for advance care planning (ACP). This qualitative descriptive study describes factors affecting SHARING Choices implementation within primary care.

**Methods:**

Semi-structured interviews or focus groups with patient/family dyads (family, friends, unpaid caregivers) and primary care stakeholders (clinicians, staff, administrators) elicited perceived barriers and facilitators of SHARING Choices implementation. Field notes and interview transcripts were coded using template analysis along the Consolidated Framework for Implementation Research (CFIR) constructs. Content analysis identified themes not readily categorized within CFIR.

**Results:**

About 22 dyads, including 14 with cognitive impairment, and 30 stakeholders participated in the study. Participants were receptive to the SHARING Choices components. Enablers of SHARING Choices included adaptability of the intervention, purposive engagement of family (particularly for patients with dementia), consistency with organizational priorities, and the relative advantage of SHARING Choices compared to current practices. Perceived barriers to implementation included intervention complexity, space constraints, workflow, and ACP hesitancy. The ACP facilitator was perceived as supportive in addressing individual and organizational implementation barriers including patient health and technology literacy and clinician time for ACP discussions.

**Conclusions:**

Patients, family, and primary care clinicians endorsed the objectives and individual components of SHARING Choices. Strategies to enhance adoption were to simplify materials, streamline processes, leverage existing workflows, and embed ACP facilitators within the primary care team.

## Introduction

Strong communication between clinicians, patients, and families is foundational in high quality, safe, and effective primary care—especially for persons with dementia. The Institute of Medicine’s report on “Dying in America” emphasized the importance of person- and family-centered care, patient-clinician communication, and advance care planning (ACP) as individuals near end of life.^1^ ACP is a communication strategy that supports adults in expressing their values, goals, and preferences for future medical care.^2^ ACP is important for persons of all age ranges and disease stages, and is especially important in the context of dementia.^3,4^ However, ACP, with or without the completion of formal advance directive documentation, remains rare within routine primary care.^5^ ACP discussions have been reported to occur among just 5% to 35% of older primary care patients,^6-10^ one study found fewer than 1% of Medicare beneficiaries discussed end of life preferences with their primary care clinician.^11^ Patient, provider, and health systems factors limit person- and family-oriented communication, including ACP, within primary care.^12^ For patients with serious illnesses such as dementia, proactive and purposeful engagement of family caregivers may improve uptake of ACP and aid in understanding of patients goals, values, and preferences for care.^13^

Scaling strategies to engage older adults and their families, including those with dementia, in collaborative communication is essential for person-centered and family-oriented primary care.^14^ Communication strategies with promise for scalability include primary care visit agenda setting,^14-16^ electronic access to visit notes^17^ and patient portals,^18^,^19^ and digital ACP and advance directives.^20^^-22^ Yet, many of these strategies remain underutilized in routine primary care due to limitations on clinicians’ time, patient and family receptivity to ACP, and continued barriers of access to electronic health records (EHR) and patient portals.^23,24^ These challenges are exacerbated when considering the distinctive communication needs of older adults, especially those with cognitive impairment.^25^,^26^

Given the complex factors inhibiting routine adoption of strategies to support person- and family-centered communication within primary care, especially for persons with dementia, inclusive interventions are warranted.^12^ Individuals and families expect primary care practices to initiate ACP and provide information and referrals for dementia needs^27^,^28^ but system factors including time, knowledge, and resources often inhibit these conversations from occurring.^29^ To address this, we developed SHARING Choices,^30^ a multi-component intervention that integrates simple communication strategies that have been demonstrated to be effective but have thus far been deployed in isolation of one another, including person-family agenda setting,^^15^,^16^^ shared family access to the patient portal, and access to a trained ACP facilitator to support primary care clinicians in completing ACP. Here, we explore the barriers, facilitators, and refinements to SHARING Choices implementation within primary care practices.

## Methods

### Design

We conducted a qualitative descriptive study to elicit perceived barriers and facilitators for delivering SHARING Choices in primary care to adults 65 years and older with and without dementia.^31^^^-^33^ Primary care stakeholders were recruited from 2 health systems in the Baltimore-Washington area between March and December 2019. The Consolidated Framework for Implementation Research (CFIR),^33^ an implementation science framework designed to guide the development, implementation, and evaluation of complex healthcare interventions, framed our evaluation.^34^,^35^ The CFIR was selected to guide the evaluation as its constructs and taxonomy support systematic categorization of the factors and conditions that contribute to important implementation success, enhancing the generalizability and replicability of our results.^33^,^36^ Research objectives were to explore: (1) receptivity to SHARING Choices; (2) perceived barriers or facilitators for implementing SHARING Choices; and (3) adaptations to support SHARING Choices implementation. The protocol (IRB00192742) was approved by a single institutional review board for the study.

### SHARING Choices

**Figure 1.**
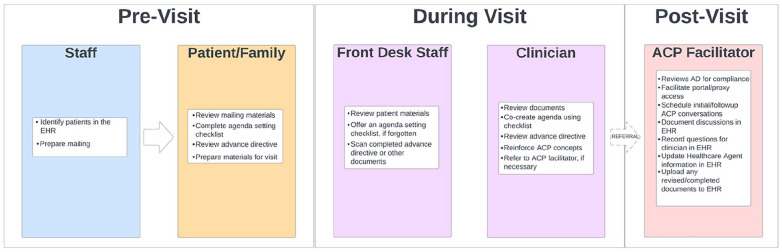
SHARING choices workflow.

The details of the SHARING Choices intervention are outlined in Figure 1.^37^ As conceptualized,^30^,^38^ front desk staff would identify patients 65 years or older with an upcoming visit from the EHR. The staff would then prepare a patient-facing postal mailing including a letter from the practice describing the program goals, orienting the patient to SHARING Choices, information on ACP and scheduling with the ACP facilitator, an agenda-setting checklist, a blank advance directive, and information on setting up shared portal access. At the visit, staff reinforces completion of the checklist, and the clinician reinforces the importance of ACP and advance directives and makes a referral to the ACP facilitator if appropriate. The ACP facilitator schedules an ACP conversation with the patient and family and assists with portal registration, as directed by the patient’s wishes prior to the patient and family leaving the practice.

### Study Sample

Using purposive sampling, we recruited 30 primary care clinicians, staff, administrators (Stakeholders) with a goal of equal representation from each health system. Stakeholders were eligible if they were 18 years or older, English speaking, and had a potential role in delivering the SHARING Choices program (ie, front desk, medical assistant, primary care clinician) or could influence adoption (ie, practice or system administrator). Stakeholders were excluded if they worked less than 2 days per week at the practice. Stakeholders provided verbal informed consent. Patient and family dyads were recruited from primary care practices operated by our health systems partners.^30^ Eligible patients were 65 years or older who regularly attended a medical visit with an adult family, friend, or unpaid caregiver (family). Patients were screened for cognitive impairment using a 6-item cognitive screening instrument.^39^ Dyads were ineligible if either the patient, their legally authorized representative, or family was unwilling or unable to complete written informed consent.

### Interviews and Focus Groups

We explored receptivity to SHARING Choices components across CFIR domains through stakeholder and dyad semi-structured interviews.^33^,^34^ For logistical reasons, we conducted 1 focus group that included 8 physicians (3001-3008) representing 2 primary care practices. Interview guides purposively explored CFIR constructs related to intervention characteristics, organizational structures and resources, characteristics of persons implementing SHARING Choices, and proposed processes for implementing SHARING Choices within routine primary care. Interviews began with an overview of SHARING Choices, affording participants time to review materials and ask questions. Interviews ranged from 30 to 90 min and were led by investigators or trained coordinators. Interviewer notes and audio recordings (if consented) were synthesized into a summary document for analysis.

### Analysis

Interview summaries were analyzed using a template analysis approach similar to Keith et al^34^ Interviews were coded by SHARING Choices and CFIR construct, with ACP being distinguished separately from the ACP facilitator role. Inductive, open coding allowed for identification of themes not readily captured by the codebook. Templates and operational definitions are available in Supplemental Appendix 1. About 2 experienced coordinators coded interview summaries with oversight from the first author between July 2020 and June 2021. Trustworthiness was addressed through triangulation of field notes, recordings, debriefing sessions, intersection of themes within and across stakeholder groups, and consensus coding among qualitative investigators. Conflicts were adjudicated by the first author. Quotes were used to contextualize and confirm interpretation.

## Results

### Participants

Table 1 summarizes the descriptive characteristics of stakeholders (n = 30) and patient and family participants (n = 22). Stakeholders predominantly identified as female (73%), white (77%), and non-Hispanic (93.3%). Patients were on average 82 years old, white (81.8%), non-Hispanic (100.0%), and had a high school education or less (50.0%). Nearly two-thirds (63.6%) screened positive for cognitive impairment. Family members were on average 70 years old and included spouses (50.0%), adult children (40.9%) or friends (9.1%); most identified as female (81.8%).

**Table 1.** Descriptive Characteristics of the Primary Care Stakeholders, Patients, and Family Interview Participants.

	Patient	Family	Stakeholder
	n (%)	n (%)	n (%)
Mean age in years (SD, range)	82.3 (8.9, 68-97)	69.3 (9.2, 51-88)	—
Gender			
Female	16 (72.7)	18 (81.8)	22 (73.3)
Male	6 (27.3)	4 (18.2)	8 (26.7)
Educational attainment			
High school or less	11 (50.0)	3 (13.6)	—
College	4 (18.2)	11 (50.0)	—
Graduate	7 (31.8)	8 (36.4)	—
Race			
Black or African American	4 (18.2)	2 (9.5)	1 (3.3)
White	18 (81.8)	18 (85.7)	23 (76.7)
American Indian or Alaska Native	0 (0.0)	1 (4.8)	0 (0.0)
Asian	0 (0.0)	0 (0.0)	5 (16.7)
Other	0 (0.0)	0 (0.0)	1 (3.3)
Ethnicity			
Not Hispanic/Latino	22 (100.0)	21 (100.0)	28 (93.3)
Relationship to patient			
Spouse	—	11 (50.0)	—
Adult child	—	9 (40.9)	—
Sibling	—	1 (4.5)	—
Friend/neighbor	—	1 (4.5)	—
6-Item cognitive screen			
All items correct	8 (36.4)	—	—
1 incorrect item	5 (22.7)	—	—
2+ incorrect items	9 (40.9)	—	—
Health system role			
Clinician	—	—	12 (38.7)
Case manager	—	—	2 (6.5)
Other staff^a^	—	—	7 (22.6)
Administrator	—	—	6 (19.4)
Other^b^	—	—	4 (12.9)

—Indicates data not applicable or not collected.

a Includes medical assistants, front desk staff, care coordinators.

b Includes patient and family advisors, community advocates, community health workers.

Table 2 presents implementation barriers and facilitators organized by SHARING Choices component and CFIR domain. Barriers and facilitators converged regarding characteristics of individual component elements, including impressions of the impact, processes, and receptivity toward ACP and the ACP facilitator, and special considerations for patients with cognitive impairment. Clinicians, patients, and family were receptive to the SHARING Choices intervention, recognized its potential for improving communication and identified adaptations to streamline integration into routine care. In the following text we summarize key findings with illustrative quotes from participants organized by the main themes emerging from the interviews.

**Table 2.** Perceived Barriers and Facilitators of SHARING Choices Implementation Categorized by the CFIR Domain and the SHARING Choices Component.

CFIR domain	SHARING choices component					
	Patient-facing postal mailing				ACP	ACP facilitator
	Letter	Agenda checklist	Shared portal access	Advance directive		
Characteristics of the SHARING choices intervention						
Facilitators						
Clinician, health system, patient, and family receptivity to the SHARING Choices components	+	+	+	+	+	+
Relative advantage of SHARING Choices over current state	+	+	+	+	+	+
Usability and acceptability of the printed materials	+	+	±			
High degree of customization available for local adaptations	+	+	+	+		+
Established structured training program for ACP facilitation (eg, Respecting Choices)					+	+
Strong evidence-based interventions		+	+	+	+	−
Normalizing ACP as part of routine practice	+			+	+	+
Mailing process allows patients and family to think about their needs before the visit, improving readiness	+	+	+	+	+	+
ACP facilitator may put patients at ease, speak in lay language, and cover elements that clinicians less comfortable with					+	+
Barriers						
Complexity of workflow/processes for intervention delivery	−	−	−	−	−	−
Some patients not comfortable with technology			−	−		+
Advance directive forms differ by state and organization and require high levels of literacy and legal literacy				−		+
Culture and language accommodations	−	−		−	−	−
Cost related to printing, mailing, and staffing the intervention	−	−	−	−		−
Outer setting (organization/health system characteristics)						
Facilitators						
Potential for cost recovery for embedded ACP facilitator role through CMS billing for ACP time by clinicians					+	+
Intervention could aid in meeting State pay for performance measures (ie, MDPCP; MIPS)					+	+
Coupling ACP with Medicare Annual Wellness visits to waive co-pay					+	±
ACP facilitator role already exists within the EHR				+	+	+
Barriers						
Clinician production pressures and short visit times		−		−	−	+
Complexity of state laws guiding advance directives				−		+
Inner setting (practice-level characteristics)						
Facilitators						
Intervention recognized as a priority for patient care			+	+	+	+
Barriers						
Time and spatial constraints within the primary care practices		−		−	−	−
Patients may be overwhelmed by or ignore postal mailing based on having limited success with mailings in the past	−	−	−	−		+
Variability of advance directive documentation in the EHR				−		+
Characteristics of individuals (characteristics of end users of the intervention)						
Facilitators						
Clinicians, patients, and family perceived value of routine ACP				+	+	+
Intervention supports explicit care partner engagement which is valued for patients with cognitive impairment		+	+	+	+	+
Patient relationships with the care team may foster uptake and comfort with ACP facilitation				+	+	+
Barriers						
Patient belief that the advance directive is not modifiable		−	−	−		+
Patient, family, and clinician hesitancy to have ACP conversations				−	−	+
Limited technology access and literacy among older adults			−	−		+
Expectations that clinicians should conduct ACP				−	−	−
Process (recommendations for implementation process)						
Facilitators						
Normalizing practices for patient portal use and ACP			+	+	+	+
Intervention could fit into existing processes and workflows for care delivery	+	+	+	+	+	+
Engagement of broader community to normalize ACP				+	+	+
ACP facilitator should be embedded within the practice and recognized as part of the care team					+	±

Abbreviations: MDPCP, Maryland Primary Care Program; MIPS, merit based incentive payment system.

− Reflects a perceived barrier to implementation.

+ Reflects a perceived facilitator to implementation.

### Receptivity to SHARING Choices

Primary care clinicians, staff, and patients and families were receptive to SHARING Choices, appreciated that it is evidence based and allowed for adaptations to meet patient and practice-level needs. “*I think it’s an excellent approach. I like the letter, the embedded staff to help with the facilitation, and the resources available, embedding it in EHR*” (Clinician: 3001). The letter was “*warm and inviting*” (Family 1031), “*a good way to initiate interaction*” (Family: 1034) and allowed them to “*emotionally plan before coming in*” (Patient: 1044). The complexity of the mailing was a concern for patients, particularly if a family member wasn’t available to help explain it.

Shared access to the patient portal was perceived as “*helpful. . . It relieves me of the time it takes to make a good decision*” (Dyad: 1056) and better than “*essentially me signing in as my mother*” (Family: 1014). Patients, but not family, expressed concerns over privacy and confidentiality that were mitigated by assuring the proxy could be changed. The agenda checklist was perceived as “*Straightforward. . .enough to open up the conversation*” (Patient: 1067) and useful because “*if you don’t have something written down, you forget. . . it jogs your memory*” (Patient: 1073). Clinicians concurred that shared access “*helps them [patient and family] to understand what’s going on*” (Clinician 2003) and that the checklist would “*work well for priming patients and families prior to the visit*” (Clinician: 2002).

Receptivity to the ACP facilitator was mixed. One family member stated, “*somebody less trained than a nurse is like a shortcut*” (Family: 1026) while a patient expressed that “*a [non-clinician] facilitator would work, perhaps even better than a physician if they do not have time*” (Patient: 1056). Clinicians perceived a benefit of having access to the ACP facilitator, “*families do not always understand what we’re trying to ask of them. . .having someone like our case manager work with them is helpful to help bridge that gap*” (Clinician: 2003). Characteristics such as empathy, tolerance, and being a people person along with being embedded within the primary care practice (improved trust) overcome patient and family concerns about the ACP facilitator.

### Relative Advantages of SHARING Choices for Normalizing ACP

The relative advantage of SHARING Choices for normalizing ACP was expressed by all stakeholders. “*I think [the ACP facilitator] is a good idea. Basically, right now we are kind of in, I would say, a little passive mode. If they show up, we do it. But this is a step forward. . . It fills in the gap to complement the service*” (Clinician: 3001). Family stated, “*clinicians should express that ACP is a part of routine care*” (Family: 1042) and “*I like the idea of having the specially trained staff, because that means that there are people who are prepared to answer to this very uncomfortable topic*” (Family: 1044).

### Practice-Level Implications and Intervention Complexity

Space, time, and workflow constraints were factors to address when embedding SHARING Choices into primary care practices. Challenges to clinician-led ACP included *“time constraints. . .right now for our patients, anybody older than 70 years, they are assigned 30 minutes, which would make it easier for us to do that. . .but for people between 65 to 70, they still qualify for [ACP], but we have only 15 minutes”* (Clinician: 2002). Another stated that “*ACP is infrequently done by physicians because of difficulty in procedures, production pressures, and education of patients and clinicians*” (Clinician: 3008). Patients concurred, “*[i]n fact the physician doesn’t have the time [for ACP]”* (Patient: 1056). Clinicians, patients, and family also agreed that “*Someone with the training and time to be able to talk about it [ACP]*” (Patient: 1056) would address this barrier.

### Considerations for Patients With Dementia

Clinicians endorsed proactive family engagement, particularly for patients with dementia. In the context of dementia, the “*most important thing is to get the caretaker involved*” (Clinician: 2002). By engaging family, the agenda checklist supported “*patient-centered conversations that can be overwhelming especially since caregivers may only be involved during crises, leading to erratic visits*” (Clinician: 3001-3007) and address communication gaps. One benefit of SHARING Choices was that “*regardless of if they are identified as having dementia or Alzheimer’s, they are still getting a letter [with mailing materials]. We’re kind of hopeful that caregivers are maybe filling those out or helping them to fill those out [checklist, advance directive], at least bringing it to the provider’s attention that they got it.”* (Stakeholder: 4001).

### Considerations for Improving SHARING Choices Implementation

Table 3 details the recommendations from patients, family, and stakeholders on ways to adapt the SHARING Choices proposed workflow, processes, technology, and infrastructure to overcome the perceived barriers to SHARING Choices implementation. Patients, family, and clinicians supported embedding ACP in routine care recommending that “*ACP should be discussed earlier before a patient is seriously ill or at the end of life*” (Dyads: 1056 and be revisited “*after a change in health*” (Dyad: 1056) and “*during their annual physical examinations*” (Dyad: 1067). These recommendations were supported by clinicians. The ACP facilitator was seen by stakeholders including clinicians as a resource to overcome literacy, legal and technology literacy, and EHR documentation issues identified by stakeholders, patients, and family as barriers to implementation. Concerns over time (ie, to conduct ACP) and cost of the intervention (ie, ACP facilitator salary) were offset by the potential for cost recovery through pay for performance measures or coupling ACP with the Medicare Wellness Visit.

**Table 3.** Stakeholder Recommendations to Adapt SHARING Choices to Improve Uptake and Implementation in Primary Care.

Construct	Adaptation to SHARING choices
Work processes	
Patient and family	• Send checklist and advance directive to patients via mail before the visit to encourage uptake
	• Develop processes and scripts to encourage patients to bring in advance directives
Clinicians	• Develop and train on scripting/systems (eg, EHR) solution for a warm handoff from clinician to the ACP facilitator
	• Design workflow to include a referral mechanism for clinicians to ACP facilitator
	• Prepare clinicians to review and reinforce the use of the checklist
	• Develop scripting to reinforce messaging about safety and confidentiality of portal, EHR
	• ACP facilitator needs to have standardized training
Care team—collaborative tasks	• Clinician to introduce the ACP process to the patient as a point of authority and trust
	• Prepare patients and clinicians to review the agenda checklist at each encounter
	• Review advance directive/ACP visit documentation at least annually
Tools and technology	• Develop/refine structures for EHR documentation of ACP conversations and advance directive completion
	• Develop “smart phrases” to standardize ACP communication in the EHR
	• Develop training to improve EHR documentation of ACP/ advance directive
	• Develop order sets for ACP billing
Organization	• Allow local adaptations to accommodate clinician, patient, and practice workflow
	• Consider alternatives to in-clinic ACP consultations given practice space constraints
Persons	• Engage practice-team to identify and refer patients to ACP facilitator
	• Develop standards, training, competencies and role descriptions for ACP facilitator
	• Engage practice leaders regularly to monitor progress
Tasks	• Multiple approaches to getting the agenda checklist to patients, including mail and having copies at reception for those who forget it
	• Develop workflows for ACP conversations annually
	• Develop workflows for revisiting goals of care with a change in status of the patient (ie, after hospitalization or new serious illness diagnosis)
Internal environment	• Identify space/accommodations for in-practice ACP facilitation (eg, space)
	• Embed ACP facilitator role within local practice teams and health system
External environment	• Leverage ACP billing codes to support ACP facilitator salary and infrastructure
	• Adhere to State/District regulations on advance directive completion
	• Develop processes to support referrals to legal or religious counsel

## Discussion

Patient, clinician, and systems-level characteristics too often inhibit patient- and family-centered communication for older adults in primary care. Clinicians, patients, and family acknowledged the potential for SHARING Choices to improve communication, particularly in supporting family engaged care and normalizing routine ACP. The family engaged communication strategies within SHARING Choices were considered particularly relevant for persons with dementia. While complex, SHARING Choices was considered flexible enough to adapt to local primary care practice contexts, resources, and was strengthened by alignment with current quality payment programs.^40,41^

The receptivity to SHARING Choices revolved around its relative advantages over the current approaches to ACP in primary care. In our study, patients and families wanted ACP introduced early and revisited regularly, especially for patients with dementia, to minimize family burden and enhance appreciation for patient’s care preferences.^42^ By normalizing ACP, SHARING Choices reduced clinicians’ concerns and supported family expectations about ACP. By routinizing ACP, SHARING Choices was advantageous for persons with dementia, by addressing ACP early thereby overcoming issues of reduced decisional capacity with cognitive decline.^42^ Similar to others, in this study we found that patients and family expected clinicians to orient patients to ACP at their first visit, initiate ACP discussions, and to routinely revisit ACP at least annually, suggesting an expectation to engage in ACP with their primary care clinician, outside of a serious illness diagnosis.^42-45^

Similar to other published studies, patients and family preferred their clinician to conduct ACP.^42,45^ Our study extends these findings suggesting that older adults were amenable to an ACP facilitator as long as the facilitator is clearly connected with the practice, working with their clinician, and had the knowledge, skills, and time to engage in ACP. Embedding the ACP facilitator within a practice could promote trust and address systemic barriers to ACP, including educating patients, privacy and confidentiality, issues with advance directive documentation and enhancing family engagement.^42,46-49^ Other benefits of the ACP facilitator included orienting the surrogate decision maker to their role in decision making at the end-of-life, navigating complex family dynamics, illuminating patient’s preferences for care, and addressing surrogate decision maker concerns. Together, this underscores the importance of close collaboration between the clinician, ACP facilitator, and the patient and family for implementation success.

Limited time, knowledge, skills, and production pressures identified as barriers to ACP, in our study are aligned with reports by others,^42,46,49-52^ and have spurred the growth of team-based models for ACP in primary care.^47^,^48^,^53^,^54^ Despite support for SHARING Choices, intervention complexity, patient preferences for clinician-led ACP, limitations of patient and family technology literacy, and information privacy concerns may limit widespread adoption of SHARING Choices. These concerns have been cited by others as barriers to uptake of patient-family shared portal access, agenda-setting, and ACP.^18,55-57^ Control in information sharing, namely controlling when, how, and who information is shared with is central to preserving patients’ autonomy.^25,58^ SHARING Choices purposively addresses this issue through the agenda checklist (eg, checking private consult). The ACP facilitator can also overcome information control concerns by educating patients and families on how to change an advance directive or proxy access and setting expectations for how a family engages in the visit and control information flow. Patients cited these strategies as important to fostering SHARING Choices adoption.

## Strengths and Limitations

Strengths of this study include the engagement of stakeholders from 2 large health systems serving diverse patient populations across a broad geographic region of the U.S. Another strength is our purposive approach to eliciting mitigation strategies from stakeholders to overcome implementation barriers and to adapt SHARING Choices prior to a large pragmatic trial.^45^ Our results are limited due to the small sample size of patient-family dyads, including those with cognitive impairment, limited racial and ethnic diversity of the participants, and purposive sampling of patients and primary care physicians from practices who had agreed to pilot SHARING Choices, and a small heterogeneous group of primary care stakeholders that engaged in the interviews.

## Conclusions

SHARING Choices was developed as a flexible approach to embedding communication strategies within primary care, especially for persons with dementia. Adaptations to mitigate SHARING Choices complexity and optimize alignment with operational priorities will enhance its strengths to engage family and normalize ACP in primary care. Future directions of this work include utilizing the barriers and facilitators identified in Table 2 will be used to support modifications (ie, as summarized in Table 3) prior to a pragmatic trial^37^ which will aim to further elucidate the impact of SHARING Choices on health system and patient outcomes and extend our understanding of barriers and facilitators to implementation.

## Supplemental Material

sj-docx-1-jpc-10.1177_21501319221137251 – Supplemental material for Perceived Barriers and Facilitators of Implementing a Multicomponent Intervention to Improve Communication With Older Adults With and Without Dementia (SHARING Choices) in Primary Care: A Qualitative Study Click here for additional data file. Supplemental material, sj-docx-1-jpc-10.1177_21501319221137251 for Perceived Barriers and Facilitators of Implementing a Multicomponent Intervention to Improve Communication With Older Adults With and Without Dementia (SHARING Choices) in Primary Care: A Qualitative Study by Kelly M. Smith, Danny Scerpella, Amy Guo, Naaz Hussain, Jessica L. Colburn, Valerie T. Cotter, Jennifer Aufill, Sydney M. Dy and Jennifer L. Wolff in Journal of Primary Care & Community Health

sj-docx-2-jpc-10.1177_21501319221137251 – Supplemental material for Perceived Barriers and Facilitators of Implementing a Multicomponent Intervention to Improve Communication With Older Adults With and Without Dementia (SHARING Choices) in Primary Care: A Qualitative Study Click here for additional data file. Supplemental material, sj-docx-2-jpc-10.1177_21501319221137251 for Perceived Barriers and Facilitators of Implementing a Multicomponent Intervention to Improve Communication With Older Adults With and Without Dementia (SHARING Choices) in Primary Care: A Qualitative Study by Kelly M. Smith, Danny Scerpella, Amy Guo, Naaz Hussain, Jessica L. Colburn, Valerie T. Cotter, Jennifer Aufill, Sydney M. Dy and Jennifer L. Wolff in Journal of Primary Care & Community Health
