# Supplementary material for: Perceived Barriers and Facilitators of Implementing a Multicomponent Intervention to Improve Communication With Older Adults With and Without Dementia (SHARING Choices) in Primary Care: A Qualitative Study
Source: J Prim Care Community Health. 2022 Nov 18;13:21501319221137251. doi: 10.1177/21501319221137251 (PMC9677296; doi:10.1177/21501319221137251)
Supplement: sj-docx-1-jpc-10.1177_21501319221137251 – Supplemental material for Perceived Barriers and Facilitators of Implementing a Multicomponent Intervention to Improve Communication With Older Adults With and Without Dementia (SHARING Choices) in Primary Care: A Qualitative Study [file sj-docx-1-jpc-10.1177_21501319221137251.docx]

**Template 1: SHARING Choices Component**

Codes for the template analysis involved breaking down feedback by the component of the SHARING Choices intervention. Initially, we did not break out Advance Care Planning as being separate from the facilitator, however, the codebook was adapted to identify feedback/perceptions on ACP directly based on the review of the first five interview and focus group summaries.

| **SHARING Choices Component** | **Operational Definition** |
| --- | --- |
| **Letter** | Perception of/or feedback on the letter from the practice. Feedback on the overall mailing process was included under the “Letter” component. |
| **Agenda Setting Checklist** | Perception of/or feedback on the agenda setting checklist. |
| **Proxy Access/Shared Access to Patient Portal** | Perception of/or feedback on the concept of proxy access or shared access to the patient portal. |
| **Advance Directive (document)** | Perception of/or feedback on the advance directive documentation. |
| **Advance Care Planning Facilitator** | Perception of/or feedback on the role of the advance care planning facilitator component of the SHARING Choices intervention. |
| **Advance Care Planning*** | Perception of/or feedback on advance care planning.  *This intervention component was added as a separate category after review of the first few interviews based on stakeholder discussion of ACP separate that was distinct from the ACP facilitator role and availability. |

**Template 2: CFIR Domains**

Selected domains of the CFIR with operational definitions adapted from the CFIRguide.org to fit with the SHARING Choices intervention and the focus. The codebook was simplified by removal of codes that were not identified in the initial review of the interview summaries.

**CFIR Domains**

The interview guides were designed to elicit perceived barriers and facilitators to SHARING Choices implementation along Domains I-IV. Adaptations to mitigate the barriers and enhance adoption of SHARING Choices and processes (Domain V) to support implementation within the broader context of the two partner health systems were also explored in Stakeholder interviews/focus groups only.

1. **Intervention Characteristics**: defined as key attributes of interventions that may influence the success of implementation.
2. **Outer Setting**: A broad domain associated with constructs such as cosmopolitanism (i.e., degree an organization is networked with other external organizations), external policies and incentives (i.e., policies and programs governing payment), patient needs and preferences, and peer pressure (i.e., competitive pressure to implement an intervention; competing organizations; competitive edge).
3. **Inner Setting**: Is a domain that is considered an active and interacting facet rather than a passive backdrop within which activities are implemented and includes constructs including structural characteristics (i.e., practice size, space), networks and communications (i.e., social networks; control of communication within the organization), organizational culture, implementation climate (i.e., absorptive capacity for change), and readiness for implementation (i.e., organizational commitment to the intervention).
4. **Characteristics of Individuals**: This domain is associated with multiple constructs that relate to the interplay between the actions and behaviors of individuals (i.e., patient, family, physician, staff), teams (i.e., local practice teams; organizational leaders), units (i.e., practices), networks, and organizations may impact the success of the implementation.
5. ***Process:** Cross-cutting domain that examines the influence of four constructs including engaging, executing, planning, and reflecting and evaluating an intervention. As this domain focuses principally on the outcomes of an implementation, it was not a primary focus of our interview guides, and thus elicited insights on how to engage practices and how to plan the implementation within each study organization.

The operational definitions for the template analysis at the construct level are summarized below and are based on the qualitative codes for the CFIR available at: [www.cfirguide.org](http://www.cfirguide.org).

| **Construct** | | **Short Description** |
| --- | --- | --- |
| **I. INTERVENTION CHARACTERISTICS** | | |
| **C** | **Relative Advantage** | Stakeholders’ perception of the advantage of implementing the intervention versus an alternative solution. |
| **D** | **Adaptability** | The degree to which an intervention can be adapted, tailored, refined, or reinvented to meet local needs. |
| **F** | **Complexity** | Perceived difficulty of implementation, reflected by duration, scope, radicalness, disruptiveness, centrality, and intricacy and number of steps required to implement. |
| **G** | **Design Quality & Packaging** | Perceived excellence in how the intervention is bundled, presented, and assembled. |
| **II. OUTER SETTING** | | |
| **A** | **Patient Needs & Resources** | The extent to which patient needs, as well as barriers and facilitators to meet those needs, are accurately known and prioritized by the organization. |
| **D** | **External Policy & Incentives** | A broad construct that includes external strategies to spread interventions, including policy and regulations (governmental or other central entity), external mandates, recommendations and guidelines, pay-for-performance, collaboratives, and public or benchmark reporting. |
| **III. INNER SETTING** | | |
| **A** | **Structural Characteristics** | The social architecture, age, maturity, and size of an organization. |
| **C** | **Culture** | Norms, values, and basic assumptions of a given organization. |
| **D** | **Implementation Climate** | The absorptive capacity for change, shared receptivity of involved individuals to an intervention, and the extent to which use of that intervention will be rewarded, supported, and expected within their organization. |
| **1** | **Tension for Change** | The degree to which stakeholders perceive the current situation as intolerable or needing change. |
| **2** | **Compatibility** | The degree of tangible fit between meaning and values attached to the intervention by involved individuals, how those align with individuals’ own norms, values, and perceived risks and needs, and how the intervention fits with existing workflows and systems. |
| **3** | **Relative Priority** | Individuals’ shared perception of the importance of the implementation within the organization. |
| **E** | **Readiness for Implementation** | Tangible and immediate indicators of organizational commitment to its decision to implement an intervention. |
| **IV. CHARACTERISTICS OF INDIVIDUALS** | | |
| **A** | **Knowledge & Beliefs about the Intervention** | Individuals’ attitudes toward and value placed on the intervention as well as familiarity with facts, truths, and principles related to the intervention. |
| **B** | **Self-efficacy** | Individual belief in their own capabilities to execute courses of action to achieve implementation goals. |
| **E** | **Other Personal Attributes** | A broad construct to include other personal traits such as tolerance of ambiguity, intellectual ability, motivation, values, competence, capacity, and learning style. |
| **V. PROCESS** | | |
| **A** | **Planning** | The degree to which a scheme or method of behavior and tasks for implementing an intervention are developed in advance, and the quality of those schemes or methods. |
| **B** | **Engaging** | Attracting and involving appropriate individuals in the implementation and use of the intervention through a combined strategy of social marketing, education, role modeling, training, and other similar activities. |
| *Adapted from <https://cfirguide.org/constructs/> | | |

**Template 3: Barriers and Facilitators for Implementation of SHARING Choices**

| **Domain** | **Operational Definition** |
| --- | --- |
| **Barrier** | A perceived challenge for implementing SHARING Choices (or its component parts) into routine practice identified by a stakeholder during the interview of focus group. This may include a negative sentiment about an intervention component, or overall guidance on challenges to implementation perceived by the individual or group.   - Statements that reflect things that might prevent success with SHARING Choices or its component. - Statements that reflect perceived attitudes or beliefs that mind hinder implementation success. |
| **Facilitator** | A person, process/task, technology, attitude, behavior, or approach perceived to support the implementation of SHARING Choices (or its component parts) into routine practice identified by a stakeholder during the interview or focus group.   - Statements that reflect perceptions of SHARING Choices that may support implementation. - Strategies to overcome a stated barrier to adoption, implementation, or sustainability of SHARING Choices or its components. |
