# Supplementary material for: Perceived Barriers and Facilitators of Implementing a Multicomponent Intervention to Improve Communication With Older Adults With and Without Dementia (SHARING Choices) in Primary Care: A Qualitative Study
Source: J Prim Care Community Health. 2022 Nov 18;13:21501319221137251. doi: 10.1177/21501319221137251 (PMC9677296; doi:10.1177/21501319221137251)
Supplement: sj-docx-2-jpc-10.1177_21501319221137251 – Supplemental material for Perceived Barriers and Facilitators of Implementing a Multicomponent Intervention to Improve Communication With Older Adults With and Without Dementia (SHARING Choices) in Primary Care: A Qualitative Study [file sj-docx-2-jpc-10.1177_21501319221137251.docx]

**Appendix 2. CONSORT Diagram for Dyad Recruitment**

**Mailed Letters n=151**

**Patients**

**Enrolled n=22 (17.19%)**

**Patient-Care Partner Dyads**

**Screened n=128 (84.77%)**

**Patient-Care Partner Dyads**

Opt-out card returned by mail to study:

n=13 Not eligible (8.61%)

12 Not accompanied

1 Too sick/hospitalized

n=10 Refused (6.22%)

1 Too busy

1 Too much trouble

8 Other/unspecified

n=22 Eligible (17.19%)

17 Consent and interview

5 Too impaired to complete screening

n=13 Not reachable (10.16%)

n=66 Not eligible (51.56%)

48 Not accompanied

1 Paid companion

3 Too sick/hospitalized

3 Companion refuses

1 Companion not reachable

1 Not available by phone

9 Other

n=27 Refused (21.09%)

22 Not interested

3 Time constraint

2 Other

**Dyad Interview**

n=22 Patient interview completed (100%)

0 Too impaired in person

n=22 Care partner interview completed (100%)
